# 

**Published:** 1889-07

**Authors:** 


### CONTENTS—JULY, 1889.—VOL. 5, NO. 1.

| | Page. |
|---|---|
| ORIGINAL COMMUNICATIONS— | |
| Puerperal Eclampsia. By H. S. Fountain, M. D., Bryan, Texas . . . . . . . . . . . . . . . . . | 1 |
| Circumcision Enforced by Law. By J. M. Vandavel, M. D., Waco, Texas . . . . . . . . . . . . . . | 7 |
| Infantile Convulsions. By E. M. D. Bridgeford, M. D., St. Lonis, Mo., . . . . . . . . . . . . . . . . | 11 |
| Pin Extracted from a Woman's Throat. By Thomas Jay Pugh, M. D., Hearne, Texas . . . . . . . . | 14 |
| Carbolic Acid in Mammary Abscess, hypodermically. By D. B. McGee, M. D., May, Texas . . . . . . | 13 |
| CORRESPONDENCE— | |
| Newsy Letter from San Antonio. By Dr. D. Berrey . | 16 |
| Youngest Baby on Record: Is it? By Dr. Sam I. Fox. | 19 |
| SOCIETY NOTES— | |
| Mississippi Valley Medical Association . . . . . . . | 19 |
| Cherokee County Medical Association . . . . . . . | 20 |
| The Charter of the Texas State Medical Association . | 21 |
| CULLINGS FROM CONTEMPORARIES— | |
| Medical Education; from *N. Y. Medical Record* . . . . | 23 |
| Digestive Ferments. Exchange . . . . . . . . . . | 26 |
| EDITORIAL— | |
| The Journal's Mission and Aims . . . . . . . . . . | 29 |
| Physicians' Mutual Benefit Association . . . . . . . | 30 |
| The Charter T. S. M. A. . . . . . . . . . . . . . . | 31 |
| A Breach of Ethics . . . . . . . . . . . . . . . . | 32 |
| MEDICAL NEWS AND MISCELLANY— | |
| A variety of items . . . . . . . . . . . . . . . . | 35 |
| BOOK NOTICES— . . . . . . . . . . . . . . . . . | 36 |
| PUBLISHERS' NOTES— | |
| Of interest to all readers . . . . . . . . . . . . . | 38 |

